# Systemic instruction of cell-mediated immunity by the intestinal microbiome

**DOI:** 10.12688/f1000research.14633.1

**Published:** 2018-12-07

**Authors:** John Grainger, Rufus Daw, Kelly Wemyss

**Affiliations:** 1 Lydia Becker Institute of Immunology and Inflammation, Division of Infection, Immunity and Respiratory Medicine, Faculty of Biology, Medicine and Health, The University of Manchester, Manchester Academic Health Science Centre, Manchester, UK; 2 Manchester Collaborative Centre for Inflammation Research, Division of Infection, Immunity and Respiratory Medicine, School of Biological Sciences, Faculty of Biology, Medicine and Health, The University of Manchester, Manchester Academic Health Science Centre, Manchester, UK

**Keywords:** microbiome, commensal, immune, gut bacteria

## Abstract

Recent research has shed light on the plethora of mechanisms by which the gastrointestinal commensal microbiome can influence the local immune response in the gut (in particular, the impact of the immune system on epithelial barrier homeostasis and ensuring microbial diversity). However, an area that is much less well explored but of tremendous therapeutic interest is the impact the gut microbiome has on systemic cell-mediated immune responses. In this commentary, we highlight some key studies that are beginning to broadly examine the different mechanisms by which the gastrointestinal microbiome can impact the systemic immune compartment. Specifically, we discuss the effects of the gut microbiome on lymphocyte polarisation and trafficking, tailoring of resident immune cells in the liver, and output of circulating immune cells from the bone marrow. Finally, we explore contexts in which this new understanding of long-range effects of the gut microbiome can have implications, including cancer therapies and vaccination.

## Introduction

The human intestine houses a tremendous quantity and remarkable diversity of microbes, including bacteria, fungi, viruses, and protozoa. Such organisms, collectively termed the gut microbiome, form complex ecosystems capable of performing a diverse array of functions that have a wide spectrum of effects on their host’s physiology and hence health ^1– 3^. Functions include those associated with digestion and nutrient status, but sensing of the gut microbiome is also understood to have profound effects on the immune system.

**Figure 1.**
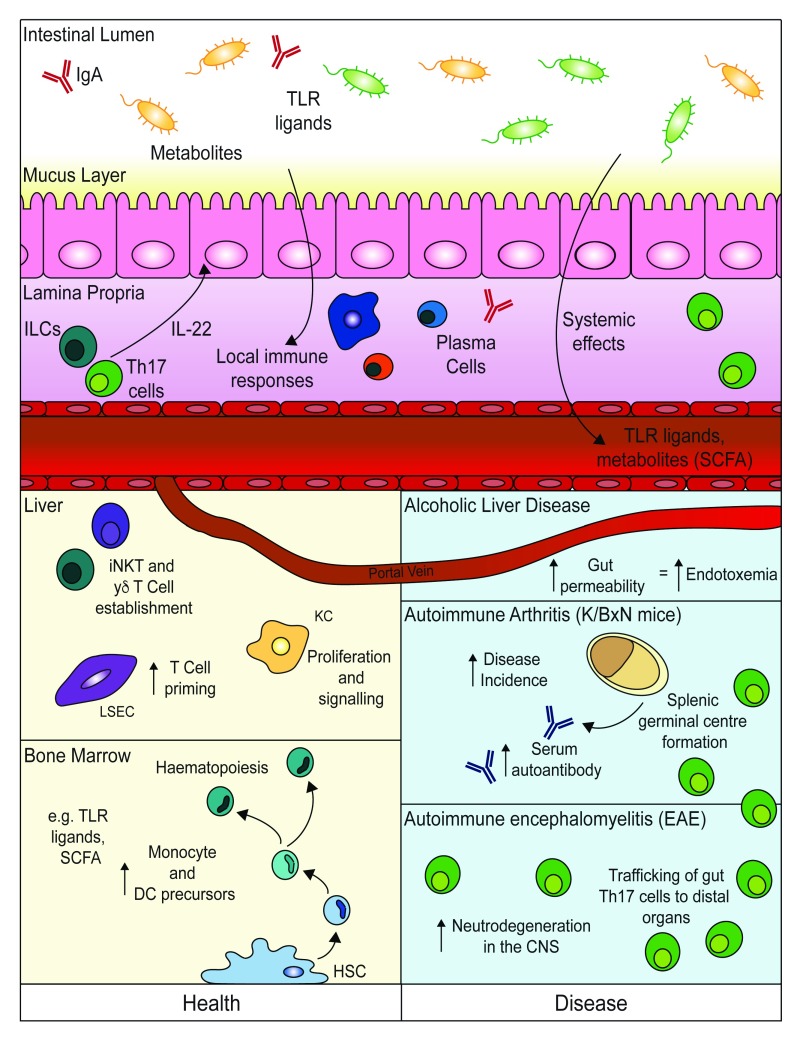
Effects of gut microbiota on systemic cell-mediated immune responses in health and disease. Much of the mucosal immune response towards the gut microbiota is focused on maintaining microbial diversity and supporting epithelial barrier function. Mechanisms include local production of immunoglobulin A (IgA) and production of the cytokine interleukin-22 (IL-22) to re-enforce epithelial barrier integrity. Even in the intact barrier, however, microbiota-derived ligands and metabolites enter into the circulatory system and impact immune populations at distal sites. These effects include tailoring of immune cell function in the liver and modulating bone marrow haematopoiesis. In disease states, the gastrointestinal barrier can become more leaky, leading to aberrant exposure to factors from the microbiome, as occurs in alcoholic liver disease. Additionally, in mouse models of T helper type 17 (Th17)-associated pathology (K/BxN arthritis and experimental autoimmune encephalomyelitis [EAE]), Th17 cells generated in response to commensal bacteria are thought to traffic from the gut and impact antibody generation and inflammation systemically. CNS, central nervous system; DC, dendritic cell; ILC, innate lymphoid cell; iNKT cell, invariant natural killer T cell; LSEC, liver sinusoidal endothelial cell; SCFA, short-chain fatty acid; TLR, Toll-like receptor.

Much of this understanding is centred on the effects of the microbiome on the development of local immune responses in the gut, particularly those related to the crucial tasks of maintaining a healthy complex microbial composition and preventing microbes from breaching the simple (one-cell-thick/unilayered) epithelium ^1, 4, 5^ ( Figure 1). For example, the production of immunoglobulin A (IgA) by gut plasma cells is important to ensure microbial diversity ^6– 8^, while interleukin-22 (IL-22) production by various lymphocyte subpopulations, including T helper type 17 (Th17) cells, γδ T cells, and type 3 innate lymphoid cells (ILC3s), stimulates antimicrobial protein release by epithelial cells ^9^.

It is increasingly recognised that the gut microbiome can influence not only these local effects on the mucosal immune system but also cell-mediated systemic immune responses ^1, 5, 10^ ( Figure 1). Evidence suggests that such systemic effects of the microbiome are intrinsically linked to both early life development of appropriate local gut mucosal immune responses towards the microbiome and their subsequent maintenance across the life course. In this regard, early life exposure to antibiotics has been linked to the development of asthma ^11^, while a decline of epithelial barrier function with age in mice leads to innate immune dysfunction in the bone marrow and peritoneum ^12^. Moreover, ongoing gastrointestinal inflammation, as occurs in inflammatory bowel disease (IBD), is associated with immune-mediated inflammation in distal organs, including the joints, skin, and eyes ^13, 14^. However, even in the healthy gut, microbial products constantly transit into the circulation ^15– 17^, and it has been suggested that in health the gastrointestinal microbiome acquires a rheostat-like function, tuning the systemic immune system ^1^.

In the 1980s and 1990s, studies using antibiotic treatment and germ-free (GF) mice highlighted effects of the microbiome on systemic immunity ^18, 19^, but owing to the recent dramatic advances in the microbiome field these systemic effects are now becoming an area of tremendous research interest. In this commentary, we will focus on three actions that are emerging as keystone mechanisms by which the gut microbiome impacts systemic cell-mediated immunity and their implications for therapies. We will specifically discuss (1) lymphocyte polarisation, trafficking, and cross-reactivity; (2) direct effects of bacterial ligands on distal organ immune cell development and function; and (3) modulation of immune cell output during haematopoiesis.

## Lymphocyte polarisation, trafficking, and cross-reactivity

At this time, perhaps the best-characterised mechanism by which the gut microbiome is known to influence systemic immune responses is via its influences on the adaptive immune system, particularly the T-cell compartment ^20, 21^. Indeed, in a number of animal models (described below), it has been established that modifying gut T cells can impact systemic disease either in a non-antigen-specific manner through bystander effects or in an antigen-specific fashion as a result of molecular mimicry by commensal factors.

In a mouse model of spontaneous autoimmune arthritis (K/BxN), GF or antibiotic-treated animals have lower serum autoantibody titres (that are associated with disease development) and ameliorated disease ^22^. This is linked to decreased germinal centre formation systemically in the animals with depleted microbiota, hence explaining the lower serum autoantibodies. When the gut of GF animals was recolonised with Th17-inducing segmented filamentous bacteria (SFB), autoimmune arthritis incidence was restored. In this setting, activated Th17 cells from the gut trafficked to the spleen, where they supported germinal centre formation and ultimately increased production of disease-mediating autoantibodies ^22^. Of note, this germinal centre formation is also dependent upon T follicular helper (Tfh) cells as in K/BxN mice it has also been shown that Tfh cells generated in the Peyer’s patches in response to SFB can transit to the spleen and support autoantibody production ^23^.

Similarly, in experimental autoimmune encephalomyelitis (EAE), a murine model for multiple sclerosis, altering the gut microbiome has been shown to modulate central nervous system (CNS) autoimmunity in a T-cell-dependent manner. In a model of spontaneous EAE, SJL/J mice raised in GF conditions were protected against developing the disease while the introduction of commensal microbiota into the gut restored susceptibility ^24^. Once again, when mice were mono-colonised with SFB, this induced Th17 cells in the gut and resulted in enhanced neurodegeneration in the CNS ^25^.

The impact of the gut microbiome on peripheral T-cell subsets can also have positive effects on inflammatory diseases. Through its capacity to induce regulatory populations, the microbiome can also support the suppression of inflammatory responses. One particularly important mechanism is via the production of short-chain fatty acids (SCFAs), including butyrate, propionate, and acetate. SCFAs are generated by the microbiota as a result of its capacity to break down fibre and are now recognised as a keystone metabolite sensed by the immune system and capable of immunomodulation ^1, 26^. SCFAs promote the differentiation of peripherally induced regulatory T cells (Tregs) and in this manner are capable of shifting the balance of effector T cells to Treg cells to limit the development of systemic inflammation ^27^. Although butyrate and propionate are dominantly restricted to the gut and hepatic portal circulation ^26, 28^, acetate can be found in the circulation, implying that SCFAs could be directly sensed by circulating T cells to alter their function.

Antigen-specific T-cell responses, as opposed to the bystander effects already described, have also been shown to play both positive and negative roles in immune-mediated diseases. Of particular note, myelin basic protein (MBP)-specific T cells can respond to structurally related microbial peptides that can result in neurodegeneration ^29^. Additionally, in autoimmune uveitis, T cells specific for self-antigens are first activated in the gut before trafficking to the eye ^30^. Conversely, myelin oligodendrocyte (MOG)-specific intraepithelial lymphocytes (IELs) were shown to transit from the gut to the CNS, where they were able to suppress neuroinflammation locally via a LAG3-dependent mechanism ^31^, again demonstrating, as is the case for the bystander effect of the microbiome on T cells, that gut commensals can be important in balancing systemic immune responses.

## Distal organ immune cell development and function

Independently of activation and T-cell trafficking from the gut-associated lymphoid tissue (GALT) to peripheral sites, resident immune cell function in organs distal to the gut can also be directly impacted by their sensing of circulating commensal-derived factors. This is particularly well exemplified by the immune populations of the liver, an organ that receives about 80% of its blood via a major tributary associated with the gut, the portal vein ^32– 34^. The composition of the immune compartment in the liver, as at other sites, is highly tailored to the specialised physiologic and immunologic requirements of the organ. Of note, the immune system of the liver, compared with that of other organs, is particularly enriched in unconventional lymphocyte populations, including invariant natural killer T (iNKT) cells and γδ T cells. These cells, which can respond to microbial lipids, are important in the protection against infections that manage to enter the circulation, but their aberrant activity can also lead to liver pathology ^33, 35^. A number of studies have implicated the gut microbiota in determining the establishment and function of these dominant cell populations.

iNKT cell numbers have been described to be positively or negatively regulated by the commensal microbiome depending on the strain of animal and type of microbiota present ^36^. Additionally, iNKT cells can exhibit functional alterations in the absence of a commensal microbiome, as they have a less-mature phenotype and are hyporesponsive to stimulation with the lipid α-galactosylceramide (α-GalCer) ^37^. A study by Li *et al.* showed that, alongside effects on iNKT cells, IL-17A-producing liver γδ T cells are also supported by the commensal microbiota ^35^. Notably, GF or antibiotic-treated animals had reduced numbers of hepatic IL-17A-producing γδ T cells; complete restoration of this population was possible through recolonisation with a complex microbiota, whilst partial restoration occurred upon the addition of *Escherichia coli* alone in a dose-dependent manner ^35^.

The liver is home to not only iNKT cells and γδ T cells but also various antigen-presenting cell populations, including dendritic cells (DCs) and the major liver-resident macrophage, the Kupffer cell (KC) ^33, 38^. These cells are responsive to microbial signals via their expression of various Toll-like receptors (TLRs) ^39^. In the early 1990s ^40^, it was suggested that KCs can recognise and respond to intestine-derived bacterial endotoxins; more recently, it has been demonstrated that KC proliferation and major histocompatibility complex II (MHC II) expression are controlled by a live gut microbiome ^41^. Indeed, aberrant changes to the gut microbiome are associated with increased hepatic inflammation, mediated partly by KC recognition of intestinal microbiota-associated molecular patterns via TLR-4/9 signalling and their subsequent upregulation of tumour necrosis factor-alpha (TNF-α) ^42^.

Though not a haematopoietic immune population, liver sinusoidal endothelial cells (LSECs) can also present antigens recognised in the sinusoidal space because of their expression of MHC I and MHC II ^43, 44^, various scavenger receptors ^45, 46^, and lymphocyte adhesion molecules such as DC-SIGN ^47^. Along with KCs, LSECs prime liver-localised CD8 ^+ ^and CD4 ^+^ T cells in response to the recognition of microbe-associated ligands passing through the sinusoids that can originate from the intestine ^44^. Thus, overall, the cellular composition and gross structure of the liver seem to co-operatively enable resident immunological subsets to respond to microbe-derived ligands derived from the intestinal microbiota.

As discussed, even in the absence of intestinal inflammation, the liver is chronically exposed to intestinally derived microbial products such as lipopolysaccharide (LPS). Owing to this baseline LPS exposure, endotoxin tolerance is observed in the liver and is associated with the priming and entrapment of tolerogenic CD4 ^+^ and CD8 ^+^ T cells by LSECs ^48, 49^ and IL-10 secretion by KCs and conventional DCs ^50, 51^. However, these immunologic subsets remain capable of responding to high LPS concentrations, and it is possible that increased LPS stimulation (greater than the baseline levels), or LPS exposure in conjunction with additional pathogen-associated molecular patterns (PAMPs)/metabolites, acts as a means to signal alterations to the commensal microbiome or intestinal barrier breach or both. This was previously proposed by Belkaid and Naik, who suggested that the liver may sense a commensal microbiome ‘molecular fingerprint’ and that changes to this ‘fingerprint’ could act as an alarm to the periphery ^10^. Perturbations to this dialogue between the gut and liver are exemplified by the pathological progression of alcoholic liver disease (ALD). ALD is associated with increased gut permeability (movement of commensal microbes outside of the gut) and in turn endotoxemia ^52^, mediated by increased ethanol consumption and commensal outgrowth ^53^, where the recognition of increased LPS titres by TLR-4 and CD14 leads to hepatic inflammation and steatosis ^54^.

It is clear that immune populations in highly vascularised organs in addition to the liver can be impacted by the gut microbiome. In particular, non-mucosal mononuclear phagocytes have been shown to have altered methylation patterns at key genes associated with type I interferon (IFN) production in GF animals, leading to impaired priming of natural killer cells in the spleen ^55^. Whether this is mediated by direct effects of microbial ligands on mature immune populations or is due to alterations in haematopoietic development (as discussed in the next section) is unclear. As in the liver, these effects are just beginning to be explored and hold much potential for understanding systemic complications associated with shifts in the commensal microbiome.

## Modulation of immune cell output during haematopoiesis

The ability of microbiome-derived ligands and their metabolites to enter the circulation allows resident bacteria in the gut to modulate the immune system from the earliest times of immune cell development during haematopoiesis ^56, 57^. Studies in the 1980s of GF animals, alongside specific pathogen-free animals treated with the antibiotic polymyxin, implicated Gram-negative commensal bacteria in promoting the development of bone marrow granulocyte-monocyte progenitor cells ^18^. In line with this dependency of granulocyte-monocyte progenitors on the gut microbiota, more recent investigations established deficiencies in differentiated myeloid cell populations in both the spleen and the bone marrow of GF mice ^56^.

Human and mouse haematopoietic stem cells express TLRs, providing a mechanism by which circulating microbiome-derived ligands could instruct haematopoiesis ^58– 60^. In humans, signalling via TLR-2 and TLR-7 directs haematopoietic differentiation towards a myeloid cell fate ^61, 62^. After myeloid differentiation, microbiome-derived ligands can also augment the release of myeloid populations from the bone marrow. Sensing of low-level changes of the TLR-4 ligand LPS, which reflects fluctuations in circulating microbial molecules after their absorption from the gut, supports the release of mature monocytes from the murine bone marrow in a CCL-2-dependent fashion ^63^.

Another mechanism by which the gut microbiome can influence haematopoiesis is via exposure to commensal-dependent metabolites. Systemic increases of the SCFA propionate, by administration in drinking water, led to alterations in bone marrow haematopoiesis characterised by enhanced DC precursor production ^64^. Gastrointestinal helminth infections can lead to alterations in the gut microbiota such that systemic SCFA levels are increased ^65^. In line with these increases in SCFA during helminth infection, DC precursors are also modulated in the bone marrow ^65^. Ultimately, this altered bone marrow output can have implications for inflammation at other mucosal sites. For example, following the administration of propionate in drinking water, the development of allergic responses in the lung is reduced. This alteration is associated with the presence of DCs that have impaired T-cell-activating capacity in the lung ^64^. This finding highlights the possibility that manipulating the factors, such as metabolites, that gut microbiota produce limits inflammation at distal sites.

## Therapeutic opportunities and future directions

As highlighted, better characterisation of the gut microbiota and understanding of its mechanisms of action on systemic immune responses hold tremendous opportunities for the development of therapeutics and also patient stratification. Two current areas of particular research interest are cancer therapy and vaccination responses ^66, 67^.

GF and antibiotic-treated mice show reduced tumour regression and impaired survival following treatment with chemotherapeutic agents compared with controls ^68, 69^, while recolonisation with specific bacterial species can lead to a restored anti-tumour efficacy ^70^. Recently, immunotherapy has revolutionised cancer treatment, particularly with regard to checkpoint blockade. In this context, T-cell pathways that are associated with regulatory checkpoints, such as PD-1 and CTLA-4, are inhibited to augment anti-tumour responses ^71^. Remarkably, the capacity of CTLA-4 blockade to have anti-tumour effects was reliant on *Bacteroides* species ^72^. Both a mouse model and studies in patients revealed that specific T-cell responses to *Bacteroides thetaiotaomicron* and *Bacteroides fragilis* correlate with the efficacy of CTLA-4 blockade ^72^. In a similar vein, *Bifidobacterium* was shown to improve melanoma control alongside PD-L1-specific antibody therapy. Indeed, in tandem, the presence of *Bifidobacterium* with PD-L1 resulted in tumour clearance ^73^.

It is becoming clear that the precise gut microbiome of an individual also has implications for the development of vaccine-mediated protection. This has perhaps been best demonstrated in GF animals or animals treated with antibiotics, where an absence or reduced gut microbiome is associated with impaired IgG and IgM responses to the seasonal influenza vaccine ^74^. This effect is dependent upon the capacity of the microbiome to act as an adjuvant via TLR-5-mediated sensing of bacterial flagellin. Oral reconstitution of GF mice with flagellated strains of *E. coli* (but not aflagellated forms) restored vaccine responses. Interestingly, this effect may be specific to certain types of vaccine, as the seasonal influenza vaccine is a non-adjuvanted vaccine ^74^. Studies of human cohorts, alongside murine models, have demonstrated effects of the microbiome on vaccine responses. For example, the relative abundance of specific bacterial species in stool microbiota of a small cohort of Bangladeshi infants was correlated with vaccine-specific IgG and T-cell proliferation towards vaccinations, including Bacillus Calmette–Guérin and hepatitis B vaccine ^75^.

Researchers are just beginning to understand the variety of mechanisms by which the gut microbiome can influence systemic immunity and the implications of this for human health. This review has highlighted three distinct types of mechanism that are already being explored ( Figure 1). Another emerging field, not discussed here, that is likely to be critical to the modulation of systemic immunity by the gut microbiota consists of interactions with the nervous system. It is well established that the microbiome is involved in instructing the nervous system, but precisely how this can lead to alterations in the peripheral immune system is less well understood ^76^. It is clear that, irrespective of the exact mechanisms, improved understanding of the key pathways and bacterial species involved in systemic instruction of the immune system holds promise to inform the development of novel therapeutic strategies to modify immune function. One such evolving strategy is faecal microbiota transplantation (FMT), in which the faecal material from a healthy donor is transferred to a patient with suspected microbial dysbiosis to restore diversity of the commensal microbiota ^77^. FMT has been used successfully to treat recurrent *Clostridium difficile* infection in patients without IBD ^77, 78^, and studies suggest that FMT may be beneficial to some patients with IBD ^79^. Whether FMT can also promote the resolution of systemic disease symptoms associated with gut inflammation and microbial alterations is just beginning to be explored, and studies are underway in diseases, including psoriatic arthritis ^80^.

Another widely promoted approach for modulating the gut microbiome is the use of probiotic therapies ^81, 82^. In rodent models, probiotics can improve systemic inflammatory disease, such as joint inflammation ^83^. However, a recent publication has suggested that, following antibiotic treatment, the use of probiotics may compromise gut mucosal recovery, demonstrating that such therapies need to be employed with caution ^84^. These early studies of microbiome-modifying therapies reveal that much future work is required to translate our rapidly advancing knowledge of how the gut microbiome impacts systemic immunity into altered patient outcomes. Even so, there is no doubt that such research does hold much promise for improving treatments in diverse disease states from cancer to autoimmunity.
